# Pure retroperitoneal natural orifice translumenal endoscopic surgery (NOTES) transvaginal nephrectomy using standard laparoscopic instruments: a safety and feasibility study in a porcine model

**DOI:** 10.1186/s12894-016-0145-7

**Published:** 2016-06-11

**Authors:** Dechao Wei, Yili Han, Mingchuan Li, Yongxing Wang, Yatong Chen, Yong Luo, Yongguang Jiang

**Affiliations:** Department of Urology, Beijing Anzhen Hospital, Capital Medical University, Beijing, 100029 People’s Republic of China

**Keywords:** models, Animal, Natural orifice translumenal endoscopic surgery/method, Retroperitoneal space, Transvaginal surgery, Nephrectomy

## Abstract

**Background:**

Among the different organs used for NOTES (natural orifice translumenal endoscopic surgery) technique, the transvaginal approach may be the optimal choice because of a simple and secure closure of colpotomy site. Pure and hybrid NOTES transvaginal operations were routinely performed via transperitoneal access. In this study, we investigate the safety and feasibility of pure retroperitoneal natural orifice translumenal endoscopic surgery (NOTES) transvaginal nephrectomy using conventional laparoscopic techniques in a porcine model.

**Methods:**

Six female pigs, weighing an average of 30 kg, were used in this study. Under general anesthesia, pure retroperitoneal NOTES transvaginal nephrectomy was conducted using standard laparoscopic instruments. Posterolateral colpotomy was performed, and the incision was enlarged laterally using blunt dissection and pneumatic dilation. A single-port device was inserted to construct the operative channel. The retroperitoneal space was created using sharp and blunt dissection under endoscopic guidance up to the level of the kidney. Dissection and removal of the kidney were performed according to standard surgical procedure, and the colpotomy site was closed using interrupted sutures. The survival and complications were observed 1 week postoperatively.

**Results:**

Our results showed that two cases failed because of peritoneal rupture. One case was successful, but required the assistance of an extra 5 mm laparoscopic trocar inserted in the flank. Three cases of pure retroperitoneal NOTES transvaginal nephrectomy were completed, and survived 1 week after the operation. In these three cases, no intra- or postoperative complications were observed.

**Conclusions:**

All findings confirmed the safety and feasibility of the retroperitoneal pure retroperitoneal NOTES transvaginal nephrectomy using standard laparoscopic instruments, which suggested the possibility of clinical application in human beings in the future.

## Background

The introduction of minimally invasive surgery has promoted the development of new surgical techniques, including the natural orifice transluminal endoscopic surgery (NOTES). The NOTES technique allows the use of hollow organs (i.e. stomach, bladder, rectum, or vagina) to access the peritoneal cavity during abdominal surgery [1, 2]. There are some theoretical advantages of this technique over open and conventional laparoscopic surgery, including avoidance of incision-related complications (wound infections, adhesions, and hernias), less postoperative pain, improved aesthetics, and a diminished immunologic impact [3, 4]. As the NOTES progress, there is a potential to make a paradigm shift in urological surgery.

Among the different organs used for NOTES technique, the transvaginal approach may be the optimal choice for female patients because of the simple and secure closure of colpotomy site. Animal studies have showed the feasibility and safety of this access route of NOTES procedures [5, 6]. Kaouk et al. also reported the use of NOTES transvaginal nephrectomy in human beings for the first time [7].

In urology, the traditional laparoscopic surgery can be performed in a minimally invasive manner using the retroperitoneal access route. Operations including lymphadenectomy for testicular cancer, nephrectomy, and adrenalectomy have been successfully conducted with this method. The benefits of this approach include less pain, less analgesic requirement, shorter convalescence, and shorter hospital stay [8].

Pure and hybrid NOTES transvaginal operations were routinely performed via the transperitoneal route. However, theoretically, the transvaginal approach to the retroperitoneal space may further reduce the invasiveness of surgery. Zorron et al. first completed excision of cyst of the kidney using flexible transvaginal retroperitoneoscopy with the assistance of two laparoscopic 5-mm trocars inserted in the left flank in human beings [9]. Moreover, robotic retroperitoneal NOTES transvaginal nephrectomy was completed in a cadaver model [10]. All of which suggested the feasibility of the retroperitoneal route in NOTES technique. In this study, we aimed to combine the transvaginal and retroperitoneal access routes to carry out pure NOTES nephrectomy in a porcine model using standard laparoscopic instruments and investigate the feasibility of this novel technique.

## Methods

### Animals

Six female pigs (Chinese experimental minipig, average weight 30 kg) were used in this study. All the pigs were got from Beijing Beiqijia Meile Farm (Beijing, China, license number: SCXK 2013-0005). All experimental procedures were complied with the Guidelines of Animal Care of Capital Medical University, and were approved by the Ethics Committee of Capital Medical University, China.

### Procedures

The animals were anesthetized by general endotracheal anesthesia, and then were secured to the operating table in a modified flank position. The animals were positioned in lateral decubitus position with abducted lower limbs. A lateral decubitus position could reduce the negative effects of the intestinal tract to make it easier to create a larger retroperitoneal working space, and the abducted lower limbs could provide space for the surgeon to operate in the vagina. The bladder was decompressed by a 12-Fr Foley catheter.

**Fig. 1:**
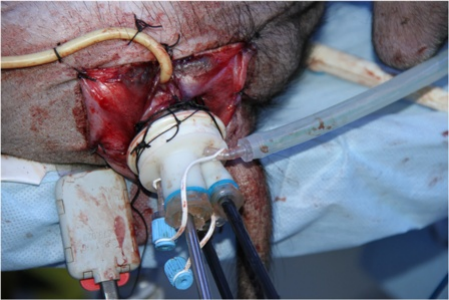
Construction of the operative channel

First, the anterior and posterior vaginal walls were incised longitudinally, and fixed to the skin with reverse sutures. Afterwards, posterolateral colpotomy was performed according to the right or left kidney. With a finger, we stretched the incision to divide the tissue. In this step, the important anatomic landmarks were the deep inguinal ring and the psoas muscle. When the finger touched upon the deep inguinal ring, blunt dissection was performed digitally to create a retroperitoneal space while maintaining contact with the psoas muscle in the posterolateral direction. Pneumatic dilation (150 mL) was used to further enlarge this cavity, and a self-designed multi-channel Triport (with our modifications) was implanted and fixed on the skin to construct the operative channel. The Triport embraced one 10-mm and two 5-mm standard channels (Fig. 1).

**Fig. 2:**
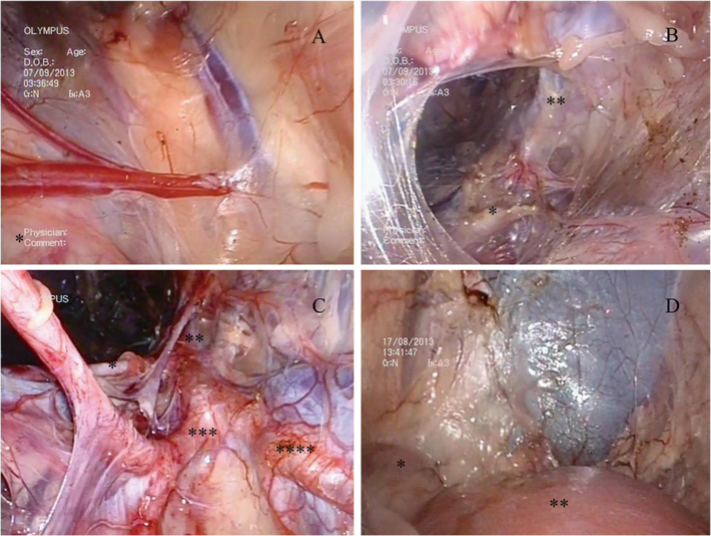
Construction of the retroperitoneal space. **a** The left side. * Rectum. **b** The left enlarged retroperitoneal space. * Medial iliac lymph nodes. ** Left external iliac vessels. **c** The right side. * Right external iliac artery. ** Aorta. *** Right internal iliac artery. **** Left internal iliac artery. **d** The left side. * Adrenal. ** Renal

The retroperitoneal cavity was insufflated with CO_2_ gas to get 15 mmHg pressures. A 5-mm hard laparoscope was subsequently inserted into retroperitoneum. With the guidance of endoscopy, the dissection of the retroperitoneal space was performed up to the plane of the kidney with two extra-long instruments. During this step, we could easily identify abdominal aorta, the iliac vessels, iliac lymph nodes, ureter, kidney, and adrenal gland (Fig. 2).

**Fig. 3:**
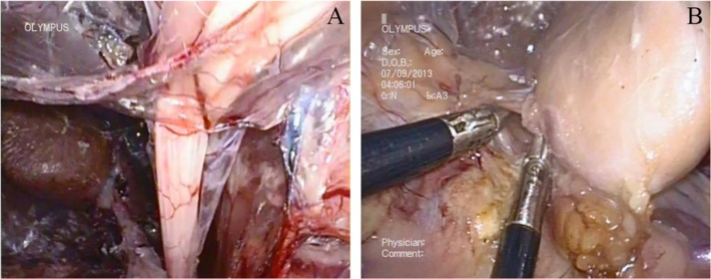
The nephrectomy procedure. **a** The treatment of the right renal vein. **b** The treatment of the left renal vein

**Fig. 4:**
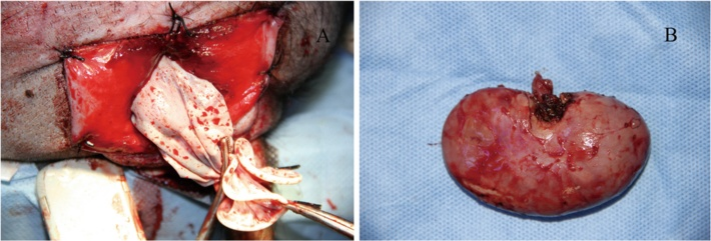
Transvaginal removal of the resected kidney, **a** Transvaginal removal of specimen. **b** Left kidney

The kidney was initially dissected using sharp and blunt techniques. Dissection was performed along the posterior aspect of the kidney, close to the psoas muscle in a medial-to-lateral direction towards the upper pole. The appropriate division of medial attachments to the kidney allowed clear visualization of the renal hilum. Finally, the superior and lateral attachments of the kidney were freed. The ureter was transected after firmly controlled with titanium clips or Hem-O-Lok clips. The renal artery and vein were also treated in the same way. In the porcine model, renal vessels could be transected directly with an ultrasonic scalpel (Fig. 3). The kidney was extracted through the existing vaginal incision in a laparoscopic retrieval bag. The incision was closed with interrupted sutures (Fig. 4). Survival and postoperative complications were observed one week after the operation.

## Results

**Fig. 5:**
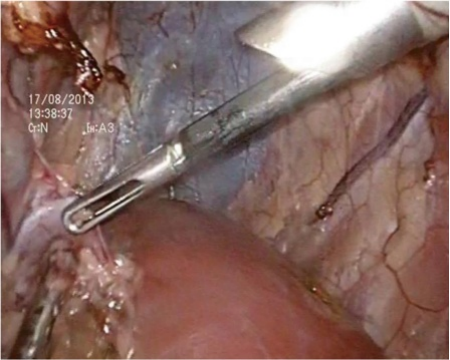
Assisted nephrectomy with an additional laparoscopic trocar in the flank

Among six cases, two failed because of peritoneal rupture. One case was successful but required the assistance of an extra 5 mm laparoscopic trocar inserted in the flank (Fig. 5). Pure retroperitoneal NOTES transvaginal nephrectomy using standard laparoscopic instruments was completed successfully in three cases, without conversion to multiport laparoscopy or open surgery (right nephrectomy [*n* = 1], left nephrectomy [*n* = 2]) (Table 1). The mean size of the removed kidneys was 10.4 cm × 5.1 cm × 3.0 cm, and the mean weight was 117 g (range, 109–125 g). After the operation, no death and postoperative complications were observed in the porcine.

**Table 1:** Summary of the cases

Sequence	Nephrectomy side	EBL (ml)	Operative time (min)	Result	Complication
1	L	–	–	Aborted	peritoneal rupture
2	R	–	–	Aborted	peritoneal rupture
3	L	100	240	Completed with additional laparoscopic port	None
4	R	100	200	Completed	None
5	L	100	195	Completed	None
6	R	100	180	Completed	None

*L* left; *R* right, *EBL* estimated blood lose

Because of improved experience and identification of the anatomic landmarks, the operation time was dramatically reduced from 240 to 180 min. No intraoperative complications, bleeding, or injury to any retroperitoneal organs occurred. Estimated blood loss was 100 mL in each case.

## Discussion

In urology, retroperitoneal laparoscopy has become the preferred option for some surgeons because of its many advantages when compared to transperitoneal access. This approach avoids intestinal disturbance and injury, allowing for quick patient recovery and short hospital stays; postoperative adhesions are minimized for the above-mentioned reasons; retroperitoneal laparoscopy avoids the negative effects of previous abdominal surgery, and the risk of wound complications are also reduced [11–13]. In addition, retroperitoneal anatomy is simpler than that of the abdominal cavity, and operative time is less than that required for the transperitoneal access [11–14]. Based on these advantages, retroperitoneal laparoscopy is widely applied in urological procedures.

In recent years, minimally invasive surgery techniques are being developed due to similar diagnostic and therapeutic effect to open surgery with less operative trauma, reduced morbidity, shorter hospital stays, minimal scarring and lower cost. Using this new technique, surgeons can employ the natural orifices such as the vagina to enter the abdominal cavity to conduct operations. This technique completely eliminates the need for abdominal incisions, and decreases the wound complications. These potential benefits of NOTES are receiving increased attention [15].

In this study, we combine the transvaginal and retroperitoneal access routes to carry out pure NOTES nephrectomy in a porcine model using standard laparoscopic instruments and investigate the feasibility of this novel technique. The aim of the study was to explore a novel laparoscopic technique and determine its feasibility and safety.

Three cases were completely successful and survived after the procedures without any operative complications such as serious bleeding. One case was successful but required assistance of an extra laparoscopic port in the flank. The first two cases failed because of peritoneal rupture. It is well known that the peritoneal membrane is very thin, peritoneal rupture commonly occurred during the step of initial dissection. It was frustrating that we failed to repair the peritoneal membrane in the two cases due to narrow space and unclear visualization. Consequently, the CO2 gas would leak into the peritoneal cavity when we tried to enlarge and maintain retroperitoneal space for operation. Finally, we have no choice but to abort the first two cases. Fortunately, with increased experience, we prevented this issue successfully. We summarized that the deep inguinal ring and psoas muscle are important landmarks when dissecting the peritoneum. Finger dissection ought to touch the deep inguinal ring first, and maintains contacting with the psoas muscle to dissect. Subsequent peritoneal dissection was simple with the guidance of video endoscope.

Another key of the procedures was the placement of the access port. To facilitated the implantation of operative instruments and prevented gas leakage, we modified the conventional soft Triport to a hard Triport, Improvement of all these measures ensured the smooth implementation of the operation. Furthermore, the length of instruments also had an effect on the operation. In this study, we used extra-long instruments (45 cm). If this technique was applied to humans, longer laparoscopic instruments would be required.

Based on successful peritoneal dissection, placement of the access port, and clear anatomic landmarks, freeing and removal of the kidney was not difficult. In this study, we found that right side of nephrectomy was easier than the left because of the rectal interference. The whole procedure was performed without any intraoperative complications such as serious bleeding or injuries to organs. No postoperative complications were observed.

Even though our research has confirmed the safety and feasibility of transvaginal laparoscopic nephrectomy through retroperitoneal access preliminarily, we are yet unable to define the explicit intraoperative and postoperative complications, given that it is the first report of pure retroperitoneal NOTES transvaginal nephrectomy using standard laparoscopic instruments. The major complications of transperitoneal NOTES transvaginal access include the risk of pelvic infection, bowel perforation, transient brachial plexus injury, and dyspareunia vaginal cuff hematoma [16, 17]. We speculate that these two accesses have similar complications.

There are still some limitations in this new technique. First, this procedure can be used only in women. However, women’s view on this new technique is still inconsistent [18, 19]. Second, it is essential to design new instruments to perform the operation successfully. Although we are able to perform the operation smoothly in the porcine model, it would require considerable improvement of laparoscopic instruments before this new technique moving on to human trials.

Pure and hybrid transperitoneal NOTES transvaginal nephrectomy in human has been reported in recent years [20–23]. Bazzi et al. successfully performed transvaginal hybrid NOTES partial nephrectomy in the porcine model by the SPIDER Surgical System [24]. The signification of retroperitoneal space through transvaginal access has also been identified in the literature [25]. Allemann et al. have performed transvaginal nephrectomy, adrenalectomy, and lymphadenectomy through retroperitoneal access in a porcine model and human cadaver in the recent years [26]. In their procedure, they completed the operation with one flexible endoscope and one laparoscopic instrument, but failed to retrieve the renal specimen due to the discrepancy between the size of the kidney and the width of the vaginal incision. Zorron et al. performed hybrid NOTES transvaginal retroperitoneoscopy to treat left renal cyst in one human case for the first time using a flexible two-channel colonoscope and two conventional laparoscopic instruments [9]. Robotic retroperitoneal NOTES transvaginal nephrectomy was also explored in a cadaver model [10]. Differing from these researchers, we performed the operation with the conventional laparoscopic technique and instruments (one 5 mm hard endoscope and two laparoscopic instruments), and removed the kidney through the transvaginal access. To our knowledge, we present the first report of pure NOTES transvaginal nephrectomy with conventional laparoscopic technique through a retroperitoneal access.

## Conclusions

Our data suggest the safety and feasibility of pure retroperitoneal NOTES transvaginal nephrectomy in a porcine model using conventional laparoscopic instruments for the first time. The potential applications of this surgical procedure include nephrectomy, adrenalectomy, and lymphadenectomy in urology, as well as surgery of the retroperitoneal organs in other field. This new technique should be further explored before be moved on human trials.

## Abbreviations

NOTES, orifice translumenal endoscopic surgery.
